# Growing up with perinatal HIV: changes in clinical outcomes before and after transfer to adult care in the UK

**DOI:** 10.7448/IAS.20.4.21577

**Published:** 2017-05-16

**Authors:** Ali Judd, Intira Jeannie Collins, Francesca Parrott, Teresa Hill, Sophie Jose, Deborah Ford, Hibo Asad, Diana M. Gibb, Caroline Sabin

**Affiliations:** ^a^ MRC Clinical Trials Unit, University College London (UCL), UCL, London, UK; ^b^ Research Department of Infection and Population Health, UCL, London, UK

**Keywords:** adolescent, young person, perinatal, HIV, United Kingdom, transition, transfer, adult care, paediatric

## Abstract

**Introduction**: With improved survival, adolescents with perinatal HIV (PHIV) are transitioning from paediatric to adult care, but there are few published data on clinical outcomes post-transfer. Using linked data from patients in the national UK/Ireland paediatric cohort (CHIPS) and an adult UK cohort of outpatient clinics (UK CHIC), we describe mortality and changes in immunological status post-transfer.

**Methods**: Participants in CHIPS aged ≥13 years by the end of 2013 were linked to the UK CHIC database. Mixed effects models explored changes in CD4 count before and after transfer, including interactions between time and variables where interaction *p* < 0.05.

**Results**: Of 1,215 paediatric participants aged ≥13 years, 271 (22%) had linked data in UK CHIC. One hundred and forty-six (53%) were female, median age at last visit in paediatric care was 17 [interquartile range, IQR 16,18] years, median duration in paediatric care was 11.8 [6.6,15.5] years, and in adult care was 2.9 [1.5,5.9] years. At last visit in paediatric care, 74% (*n* = 200) were on ART, increasing to 84% (*n* = 228, *p* = 0.001) at last visit in adult care. In the 12 months before leaving paediatric care, 92 (47%) had two consecutive viral loads >400 copies/mL or one viral load >10,000 copies/mL, and likewise 102 (52%) in the 12 months post-transfer (*p* = 0.79). Seven (3%) people died in adult care. In multivariable analysis, CD4 declined as patients approached transition with a greater decline in those with higher nadir CD4 count (mean rates of decline of 3, 13, 15, 30 cells/mm^3^ per year for those with nadir CD4 < 100, 100–199, 200–299 and ≥300 cells/mm^3^, respectively). Post-transition, CD4 continued to decline in some groups (e.g. black males, −20 (−34, −5) cells/mm^3^ per year post transition, *p* = 0.007)) while it improved in others. Overall CD4 was higher with later year of birth (14 (7, 21) cells/mm^3^ per later year). There was no effect of age at transfer or changing hospital at transfer on CD4.

**Conclusions**: Our findings suggest that CD4 in adolescents with perinatal HIV in the UK was declining in the period before transition to adult care, and there was some reversal in this trend post-transfer in some groups. Across the transition period, CD4 was higher in those with later birth years, suggesting improvements in clinical care and/or transition planning over time.

## Introduction

Adolescence is recognized as a critical period for developing patterns of self-management and laying the foundations for good health in adulthood. The rapid biological and psychosocial changes that take place during this period affect every aspect of the lives of adolescents [1], and thus it is an important stage at which to deliver supportive healthcare services. However, many studies have shown that health outcomes for chronic conditions are worse in adolescents than in children or adults; these poorer outcomes appear to be linked to poorly planned transition from paediatric to adult care [2–9]. This issue is pertinent to perinatal HIV, as around 1.8 million children were living with HIV globally in 2015 [10], and children are entering adulthood in increasing numbers. To date, few studies have investigated the effect of transition from paediatric to adult care on health outcomes in the field of HIV.

Emerging data suggest high mortality and morbidity in adolescents with perinatal HIV [11]. The World Health Organization reported that HIV had become the eighth leading cause of death for all adolescents worldwide by 2012 [12], and the fourth leading cause of disability-adjusted life-years lost, with broadly equal impact across males and females [1]. These findings were consistent with the Global Burden of Disease (GBD) Study which showed that HIV rose from the 101st cause of adolescent disability-adjusted life-years lost in 1990 to the 6th highest cause in 2013 [13]. Findings from the USA have described alarmingly poorer outcomes among adolescents generally, compared to older adults, at every step of the treatment cascade, with one study suggesting that only a third of young people with perinatal or horizontal HIV receiving HIV care in the USA were virologically suppressed [14,15]. The HIV Research Network based in the USA reported that 20% of youth aged 21 years receiving HIV care were lost to follow-up within one year [16]. In the UK and Europe, studies of young people with perinatal HIV suggest similarly poor outcomes, with increased risk of mortality [17], care disengagement [18], and treatment failure [19,20].

Paediatric HIV is a rare disease in the UK and Ireland, but people with perinatal HIV comprise one of the most mature national cohorts of adolescent and adult survivors globally [21]. By the end of 2014, 1,907 children with HIV living in the UK and Ireland had been followed through the Collaborative HIV Paediatric Study (CHIPS) cohort, of whom 644 (34%) had moved to adult care. Transition to adult care generally does not happen at a defined time point in the UK; rather, it is tailored to the individual and their specific needs [22]. Young people move from approximately 60 paediatric outpatient clinics across the UK to adult care, with some adult clinics having “adolescent-friendly” services. The UK has guidelines on transition, both for chronic diseases generally and also specifically for HIV [22,23], but it is not clear if they have led to improvements in health outcomes.

Few studies are able to directly observe patients across the paediatric and adult care pathway and compare outcomes pre- and post-transition. In this study, we linked individual patient data for children and young people with perinatal HIV across two cohorts, the national CHIPS paediatric cohort, and the UK Collaborative HIV Cohort Study (UK CHIC), a large adult cohort of outpatient clinic attenders. We describe mortality, antiretroviral treatment (ART), CD4 and viral load status pre- and post-transfer and, for the first time in a transition study, model CD4 change over the transition period and investigate predictors of change pre- and post-transition.

## Methods

The CHIPS and UK CHIC cohorts have been described elsewhere [24,25]. In brief, the National Study of HIV in Pregnancy and Childhood (NSHPC) collects reports of all infants born to HIV-infected women and all children aged <16 years diagnosed with HIV infection (regardless of country of birth) in the UK and Ireland. Subsequent follow-up information is collected annually through CHIPS, and ceases when a person transfers from paediatric to adult care, which happens at median of 17 years of age [26]. Patients transferring to adult care can choose the adult clinic which they wish to attend, although most paediatric clinics have a “partner” adult clinic which is usually the default choice for a patient. UK CHIC is a multicentre cohort of HIV-positive adults aged ≥16 years attending one of 21 collaborating adult outpatient clinics across the UK. Participating UK CHIC clinics each submit a dataset of routinely collected clinical information (including basic demographics, CD4 counts, viral loads, ART use, clinical outcomes and laboratory markers of ART toxicity and comorbidities) annually; these datasets are collated across study sites to form the study database. Both studies have National Health Service (NHS) Research Ethics approval.

Records of children and young people participating in CHIPS aged ≥13 years by the end of 2013 who were documented as having transferred to adult care were linked to records in the UK CHIC database, using date of birth, initials and sex, and subjected to further checks across a broader range of fields (e.g. ethnicity, clinic and Soundex (an indexing system which encodes surnames)) for confirmation. The last date in paediatric care was defined as the last date of a visit, laboratory marker or ART change in a paediatric clinic, and the first date in adult care was defined similarly. The following key outcomes in paediatric compared to adult care were compared: (i) proportion of the sample prescribed ART at last follow-up visit in paediatric and adult care; (ii) median CD4 count at 12 months pre- and post-transfer (individual 12 month pre- and post-CD4s were defined as the measures taken nearest to but within ±6 months of these time points); (iii) proportion with CD4 < 200 cells/mm^3^ at least once in the 12-month periods pre- and post-transfer; (iv) proportion with two consecutive viral loads (≤6 months apart) >400 copies/mL or one viral load >10,000 copies/mL in the 12 month periods pre- and post-transfer in those on ART for ≥6 months; and (v) proportion with two consecutive viral loads (≤6 months apart) >400 copies/mL or one viral load >10,000 copies/mL in the 12-month periods pre- and post-transfer (no ART criterion). Wilcoxon matched-pairs signed-ranks and McNemar’s tests compared medians and proportions, respectively. All analyses were conducted using STATA version 14 (College Station, Texas, USA).

Deaths in adult care were also described. The UK CHIC Study obtains information on deaths through clinic reporting and through linkage to Public Health England’s national HIV surveillance datasets. These surveillance datasets have good ascertainment of mortality as they are supplemented by the Office for National Statistics mortality registry for deaths occurring under the age of 65, as well as relying on clinician reporting.

Changes over time in CD4 count before and after transfer to adult care were explored using mixed effects models, allowing for multiple measures per person (including all available data, assuming that the model was robust to gaps between measures). The transfer date was defined as the last date in paediatric care. Gaps between the end of paediatric and the beginning of adult care were described but in the model were included in adult care. Time before and after transfer was modelled as a linear spline with one knot at transfer where time was set to zero. Years before and after transfer were both categorized as positive numbers around zero (e.g. 1 year pre-transition was categorized as +1, as was 1 year post-transition), to avoid having to present the results of negative main effects coefficients combined with negative interaction coefficients. Person-level random effects were included for intercept and slopes before and after transfer (unstructured covariance matrix). Models were adjusted for demographic characteristics (sex, ethnicity, born abroad, year of birth), nadir CD4 cell count in paediatric care, and suppressed viral load <400 copies/mL (yes/no/missing) within 6 months of the CD4 measurement, and time before/after transfer. Interactions between time and other variables, including age at transfer out of paediatric care, and change of hospital at transfer, were included if the interaction *p* < 0.05. In a sensitivity analysis, we included an indicator for time since previous CD4 (<1 year versus ≥1 year).

## Results

Of 701 CHIPS participants aged ≥13 years by the end of 2013 who had transferred to adult care, 271 (39%) were linked to a corresponding UK CHIC record. Characteristics of the 271 patients in both datasets are presented in Table 1. A total of 146 (53%) were female, nearly all were recorded in CHIPS as having perinatal HIV (93%), 80% had black ethnicity (of whom 99% were black African) and three-fifths (61%) were born outside the UK or Ireland, for whom median age at presentation in the UK was 9 years [interquartile range (IQR) 4, 12] years. Median age at last visit in paediatric care was 17 [16,18] years, and median age at last follow-up in adult care was 20 [19,23] years. The median duration of follow-up in paediatric care was 11.8 [6.6, 15.5] years. The median gap between last paediatric care visit and first adult UK CHIC clinic visit was 2.4 [1.0, 4.4] months (17 people had a gap of ≥12 months), and median duration of follow-up in adult care was 2.9 [1.5, 5.9] years, giving a median total duration of follow-up in HIV care of 15.4 [10.6, 19.3] years. Patients had median 3.0 [2.3, 3.8] CD4s and 2.9 [2.0, 3.6] viral loads per year in paediatric follow-up; and 3.4 [2.6, 4.0] viral loads and 3.2 [2.3, 4.6] CD4s per year in adult follow-up. At last follow-up, 86 (32%) patients had Centers for Disease Control and Prevention (CDC) Class C (AIDS defining) diagnosis [27], of whom 9 had their first CDC C event in adult care.

**Table 1.** Characteristics of CHIPS participants with perinatal HIV transitioning to adulthood in the UK and with linked data in the UK CHIC cohort (*n* = 271)

	*n* (%) /median [IQR]
**Sex (female)**	146 (53%)
**Perinatal HIV infection^a^**	251 (93%)
**Ethnicity**	
Black	213 (80%)
White	28 (11%)
Other	25 (9%)
**Born outside UK/Ireland**	163 (61%)
**Year of birth**	
Up to 1989	75 (28%)
1990–1994	160 (59%)
1995–1997	36 (13%)
**Ever taken ART**	246 (91%)
**Age started ART**	9.8 [5.9, 13.1]
**Age at last paediatric care visit (years)**	17 [16,18]
**Age at last follow up in adult care (years)**	20 [19,23]
**Duration of paediatric care follow up (years)**	11.8 [6.6,15.5]
**Duration of adult care follow up (years)**	2.9 [1.5,5.9]
**Gap between paediatric and adult care (months)**	2.4 [1.0,4.4]
**Duration of total follow up (years)**	15.4 [10.6,19.3]
**Changed hospital at transfer**	129 (48%)
**Nadir CD4 cell count in paediatric care**	194 [90, 281]
**Ever CDC Class C (AIDS) diagnosis**^b^	86 (32%)

CHIPS, Collaborative HIV Paediatric Study; UK CHIC, United Kingdom Collaborative HIV Cohort Study; IQR, interquartile range; ART, antiretroviral therapy; CDC, Centers for Disease Control and Prevention ^a^For the rest, 8 blood transfusion, 12 unknown. ^b^77 had first AIDS event in paediatric care.

**Figure 1.**
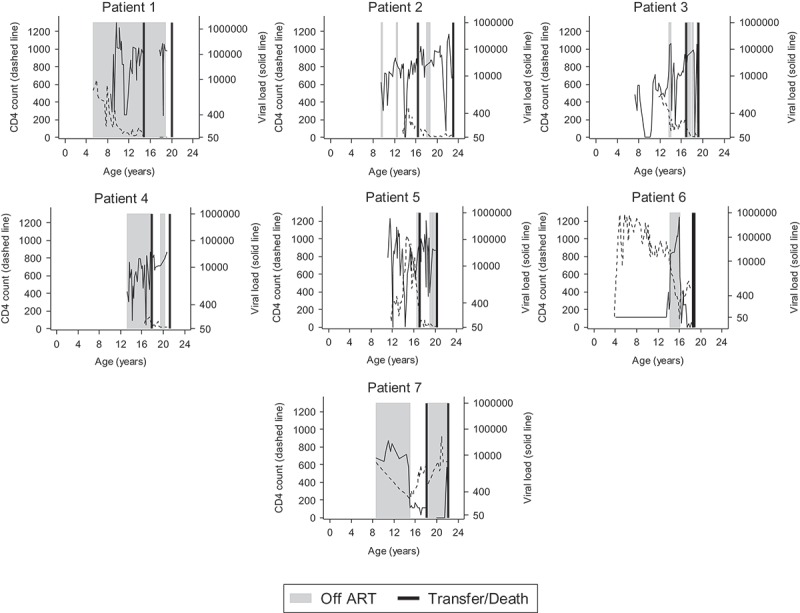
CD4, viral load, age at transfer and death, and periods where no ART was prescribed, for 7 patients who died.

Seven people (3%) died in adult care, at median age 20 [19,22] years. Six of these seven had a previous CDC C event, and of these two had their first event in adult care. Six of the seven were born outside of the UK. Causes of death were advanced HIV (*n* = 3), leukoencephalopathy (*n* = 1), renal failure (*n* = 1) and pulmonary tuberculosis (*n* = 1), and one unknown cause of death. Figure 1 presents the individual viral load and CD4 count trajectories for these seven patients, including periods where ART was not prescribed. The CD4/viral load trajectories of the seven were similar, with all experiencing CD4 decline and increasing viral load prior to death; five of the seven also had periods when they were not receiving ART prior to death according to clinic records. Most of these seven continued to access care fairly regularly after transition, with a median of 10 viral loads and 9 CD4 cell count measurements between transition and death, over a median of 3 years post-transition.

Table 2 presents key outcomes pre- and post-transfer to adult care. At last visit in paediatric care, 200 (74%) people were on ART, with this number increasing to 228 (84%, *p* = 0.001) at last follow-up in adult care. Median CD4 counts 12 months prior to leaving paediatric care and 12 months after starting adult care were 465 [288, 668] cells/mm^3^ and 460 [290, 670] cells/mm^3^, respectively (*p* = 0.38). In the 12 months before leaving paediatric care, 21% had at least one CD4 count <200 cells/mm^3^, and 23% (*p* = 0.39) in the 12 months after starting adult care. Among those prescribed ART for at least six months, 28% and 29% (*p* = 0.85) had confirmed viremia >400 copies/mL or one viral load >10,000 copies/mL in the 12 months pre- and post-transfer, respectively. When including all patients, irrespective of ART status, 47% and 52% had confirmed viremia >400 copies/mL or one viral load >10,000 copies/mL in each period respectively (*p* = 0.12). There were no significant differences in key characteristics (including sex, black ethnicity, born outside UK, ever taken ART, age started ART) between those with CD4/VL outcomes in both paediatric and adult care and those with missing data (data not shown).

**Table 2.** Key outcomes in paediatric and adult care (*n* = 271)

	Paediatric care	Adult care	
	*n* (%) /median [IQR]		*p* value
**Prescribed ART at last follow-up**	200 (74%)	228 (84%)	0.001
CD4 count 12 months pre/post transfer (*n* = 204*)	465 [288,668]	460 [290,670]	0.38
CD4 < 200 cells/mm^3^ at least once in 12 months pre/post transfer (*n* = 195*)	41 (21%)	45 (23%)	0.39
Two consecutive VL >400 copies/mL or one VL>10,000 copies/mL in 12 months pre/post transfer for those on ART for at least 6 months (*n* = 131*)	37 (28%)	38 (29%)	0.85
Two consecutive VL >400 copies/mL or one VL>10,000 copies/mL in 12 months pre/post transfer irrespective of ART status (*n* = 196*)	92 (47%)	102 (52%)	0.12

IQR, interquartile range; ART, antiretroviral therapy*n = number with paediatric and adult care data.

Univariable and multivariable predictors of change in CD4 count over time are shown in Table 3. In the multivariable model, CD4 was higher (pre- and post-transition) in those with higher nadir CD4. However, there was a decline in CD4 as patients approached transition, with a greater decline in those with higher nadir CD4 (mean declines of 3, 13, 15, 30 cells/mm^3^ per year prior to transfer for those with nadir CD4 <100, 100–199, 200–299 and ≥300 cells/mm^3^, respectively). There was no difference in the rate of change in CD4 count post-transfer according to nadir CD4 (interaction *p* = 0.48). In the period prior to transfer, CD4 was lower on average in females than males (−41 (−85, 2) cells/mm^3^; *p* = 0.06), with no evidence for a difference in the rate of change in CD4 by sex leading up to transition (*p* = 0.10). Although prior to transfer CD4 was higher amongst those of non-black ethnicity, this difference was non-significant (30 (−26, 86) cells/mm^3^; *p* = 0.30) and the rate of change did not differ by ethnicity leading up to transition (*p* = 0.28).

**Table 3.** Univariable and multivariable predictors of CD4 count change during transition to adult care

	Univariable^c^			Multivariable (*n* = 262^b^)		
Predictor	Coefficient	95% CI	*p* value	Coefficient	95% CI	*p* value
Constant				154.0	65.6, 242.5	0.001
*Main effects*:^a^						
Time before transition (per 1 year before transition)	9.2	1.7, 16.8	0.016	2.5	−9.5, 14.5	0.681
Time after transition (per 1 year after transition)	−2.3	−12.7, 8.0	0.658	−19.6	−33.8, −5.4	0.007
Female	−19.2	−72.4, 34.0	0.479	−41.1	−84.5, 2.3	0.064
Non-black ethnicity	−80.6	−146.7, −14.4	0.017	29.7	−26.2, 85.6	0.298
Born abroad	−3.5	−58.5, 51.6	0.902	−0.5	−43.7, 42.8	0.983
Year of birth (per 1 year increase)	20.0	11.5, 28.5	<0.001	13.7	6.7, 20.8	<0.001
Nadir CD4 cell count (per 10 cells/mm^3^ increase)	7.8	6.4, 9.3	<0.001	5.9	4.3, 7.4	<0.001
Viral suppression (time updated)						
Non-suppressed, ≥400 copies/mL	0.0			0.0		
Suppressed, <400 copies/mL	131.6	122.4, 140.7		134.5	125.2, 143.8	
Missing	58.9	15.6, 102.3	<0.001	60.6	16.2, 105.1	<0.001
*Interactions with time before transition (per 1 year):*						
Nadir CD4 cell count (cells/mm^3^)						
0–99	-			0.0		
100–199				10.1	−5.5, 25.5	
200–299				12.5	−2.9, 27.9	
300+				27.7	9.0, 46.4	0.038
*Interactions with time after transition (per 1 year):*						
Female				24.0	6.0, 41.9	0.009
Non-black ethnicity				34.1	11.6, 56.6	0.003

CI, confidence interval **^a^**Main effects can be considered in isolation (i.e. ignoring interactions) when all other variables are equal to 0 (continuous) and notably at transition or equal to the baseline category (categorical). ^b^Number of participants. ^c^Univariable models number of participants, *n* = 271 except for non-black ethnicity (*n* = 266), born abroad (*n* = 266), nadir CD4 cell count (*n* = 269).

After transition, sex and ethnicity influenced the rate of change in CD4; assuming independent effects (tested by fitting a 4-level categorical variable, data not shown), CD4 declined among black males (−20 (−34, −5) cells/mm^3^ per year post transition; *p* = 0.007), remained relatively stable among black females (4 (−9, 18) cells/mm^3^ per year; *p* = 0.51), and increased in white males (15 (−8, 37) cells/mm^3^ per year; *p* = 0.22) and white females (39 (17,60) cells/mm^3^ per year; *p* <0.001). After controlling for other factors, overall CD4 counts were higher among people born in later calendar years (14 (7, 22) cells/mm^3^ per later year), and among those with suppressed viral load compared to those with non-suppressed viral load (135 (125, 144) cells/mm^3^). There was no association between CD4 count and having been born abroad (*p* = 0.98). There was no evidence for an interaction between time after transition and either changing hospital at transfer (*p* = 0.23) or age at transfer (*p* = 0.49), indicating that the rate of change in CD4 post-transfer did not differ according to these factors.

**Figure 2.**
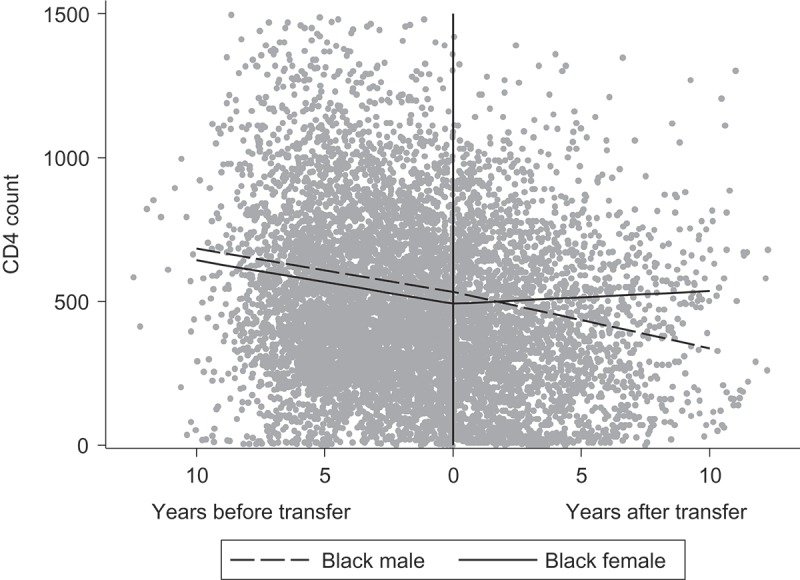
The association between sex and CD4 slopes over time for a hypothetical patient with the following characteristics: black ethnicity, born in the UK in 1991, nadir CD4 of 207, transferred to adult care at 17.5 years (sample means), no hospital change at transfer.

Figure 2 shows the association between sex and CD4 slopes pre- and post-transfer, for modelled patients of black ethnicity, born in the UK, with a nadir CD4 of 207 cells/mm^3^ and an age at transfer to adult care of 17.5 years (sample means), with no change of hospital at transfer. As transition approached, slopes declined for both males and females, and values were slightly lower for females. However after transition, the decline in CD4 for males continued, whilst for females it levelled off.

In paediatric care, 2% of CD4 measures followed a gap of ≥1 year since the previous measure; in adult care this went up to 4%. There was no evidence for an effect of time since last CD4 (< versus ≥1 year) (*p* = 0.13) and other model coefficients changed very little when this term was included.

## Discussion

In our study of young people with HIV who had been followed in our national paediatric cohort and subsequently transferred to adult care, we identified 271 in a cohort of adults attending outpatient clinics in the UK. The median duration of follow-up in paediatric care was 12 years, and adult care 3 years. A key advantage of our analysis was having a complete national paediatric dataset, from which we could link to patients attending adult clinics participating in UK CHIC.

We found no difference overall in the median CD4 count at either 12 months prior to leaving paediatric care (465 cells/mm^3^) or 12 months after starting adult care (460 cells/mm^3^), and the proportion with at least one CD4 count <200 cells/mm^3^ in the 12 months pre- and post-transfer was similar, at 21% and 23%, respectively. This was much higher than a figure of 10% of patients in UK CHIC overall with CD4 count <200 cells/mm^3^ at any time in the 24 months prior to their last visit in 2013 (S Jose, personal communication). Post-transfer CD4 in our study was similar to a mean of 413 cells/mm^3^ reported from a group of 46 young adults (7 with perinatal HIV) aged 17–24 years, 48 weeks after starting adult care in Chicago [28]. Another US study of 50 young adults (19 with perinatal infection) with a median age of 28 years (older than our study), who were transitioning to adult care in Baltimore, also reported no difference between the CD4 count 12 months pre- and post-transfer (median 347 cells/mm^3^ vs. 351 cells/mm^3^, respectively) [29]. Interestingly, our findings were also not dissimilar to a median CD4 change of 374 cells/mm^3^ at 48 weeks from a baseline CD4 count of 170 cells/mm^3^ among 31 children and adolescents aged 9–19 years with perinatal HIV infection starting ART for the first time in a community-based ART programme in Cape Town, South Africa [30]. Our CD4 findings suggest that post-transition, PHIV are in worse health than other adults with HIV in the UK, and reasons for this need further exploration. However, the relatively short gap between the end of paediatric care and the start of adult among these young people, coupled with no difference in median CD4 count pre- and post-transfer, is reassuring.

In our study although the proportion who were prescribed ART increased after transition, this could in part have been driven by a move towards universal ART in recent years [31]. However, the proportion with two consecutive viral loads greater than 400 copies/mL or one viral load >10,000 copies/mL in the 12 months pre- versus post-transition did not change (47%, 52%). This is substantially higher than the 10.2% with confirmed viral rebound reported in the broader UK CHIC population of adults starting ART for the first time [32]. Only around half of young adults in our study had a continually suppressed viral load pre- and post-transition, and the viral load trajectories of those who died after transfer to adult care indicated poor ART adherence. In the Netherlands, among 59 adolescents transitioning to adult care at median 19 years of age, around 26% had two consecutive viral loads greater than 400 copies/mL at 12 months post-transition, lower than the proportion reported in our study, although over a shorter period of follow-up [33]. In the Baltimore study, 57% of young adults had a suppressed viral load (<400 copies/mL) at 12 months post-linkage to adult care [29]. These findings are in contrast to 76% viral suppression (<40 copies/mL) at last follow-up reported from 67 adolescents engaged in a transition programme and transferred to adult care in Northern Thailand [34].

Trends in CD4 count among adolescents during the transition period varied by key socio-demographic and clinical characteristics, as well as by whether patients were pre- or post-transition. First, CD4 was higher both pre- and post-transition in patients with higher nadir CD4 compared to lower nadir CD4. This trend is consistent with current HIV treatment guidelines [35], and similar findings have been reported in studies of adults with HIV [36]; for example, among HIV seroconverters with viral suppression, the absolute CD4 count attained after ART start was highly dependent on both baseline and nadir CD4 counts [37]. However, the CD4 count declined in most groups of people as they approached transition, with a greater rate of decline in those with a higher nadir CD4 count. Declines in CD4 prior to transition may be reflective of growing autonomy and worse adherence during adolescence, and may themselves precipitate transfer to adult care. Second, after transition, the CD4 count continued to decline in black males, but remained constant in white males and black females, and increased in white females. This finding warrants further investigation, especially considering that the majority of people in our study were black African, and just under half of participants were male. An analysis of adults attending HIV clinics in England, Wales and Northern Ireland reported that black African people had higher risk of loss-to-follow-up than other ethnic groups [18]. In the Netherlands study, there were no differences in virological failure by gender [33], but other studies have not reported any gender differences.

Third, CD4 was higher in patients with later birth years. The majority of patients in our study were born pre-1996, so before the combination therapy era, and although some may not have presented for care until after 1996, this finding gives an indication of how improvements in treatment over time have improved clinical outcomes, as shown elsewhere [21,38,39]. It may also indicate improved transition planning in more recent calendar year periods. Fourth, those with suppressed viral load had substantially higher CD4 counts than those with non-suppressed viral load, highlighting the continued importance of adherence to ART in this population. Fifth, we found no effect of age at transfer, or having changed hospital at transfer, on CD4 after transition. In the Netherlands study, the average age at transition was 18–19 years, and this age group was also associated with the highest risk of virological failure [33]. The lack of effect of age at transfer in our study may be indicative of clinics trying to ensure that transition planning is developmentally appropriate and that the point of transfer is tailored to the individual person’s needs, as recommended by UK transition guidance, rather than occurring at or by a specific threshold as is practice in other countries [22,29,33,40]. However half of our cohort transitioned between the ages of 16 and 18 years, and so we do not know if outcomes may have improved if transition had been delayed until young people were in their 20 s. Additionally, the finding that changing hospital at transfer did not affect CD4 post-transition may again suggest a joined up approach between paediatric and adult care where a change in hospital does occur.

Our study has several limitations. The UK CHIC study does not include all adults receiving care in the UK, and so we were not able to link all young people who were documented in CHIPS as having transferred to adult care. In turn we were unable to measure loss-to-follow-up or mortality in those not linked to UK CHIC, as those who had moved to adult care could have been attending other non-UK CHIC clinics or could have died prior to an adult care visit. We also did not focus on any gaps between paediatric and adult care, because visit frequency in the UK depends on where a patient is on their treatment pathway as well as their CD4 level, and our patients were an “in treatment” group by virtue of them being linked between the two datasets. We are currently in the process of gaining consent to follow all PHIV into adult care in the UK, and plan subsequent analyses which can measure loss-to-follow-up and care gaps. However mortality ascertainment in UK CHIC was good, as data on deaths are linked with national mortality registers and are also reported by participating clinics. Adherence data were not available, and so we could only investigate the role of viral load as a predictor of CD4 count change. Additionally, the UK CHIC dataset does not have complete reporting of hospitalizations, a potentially important measure of the success of transition. Research in several disease areas has found poorer attendance and increases in avoidable hospitalization post-transfer into adult care, neither of which was measurable in our study [3,41]. Finally, the CHIPS and UK CHIC datasets are largely focussed on clinical and biological markers of HIV, and thus we were not able to consider the potential role of other factors, including cognition and mental health. Poorer outcomes may be due to psychosocial issues as well as biological factors, including cognitive and mental health issues [42–44], stigma and discrimination [45], HIV disclosure [46] and parental loss [47]. This limits the clinical message of our findings, and further exploration of the role of a wider set of factors is required using in-depth cohort data.

## Conclusions

Our findings suggest that CD4 in patients with perinatally acquired HIV was already declining in the period before transition to adult care, and that after transfer to adult care there was some reversal in this trend in some groups. Post-transition, CD4 declined in black males, but remained stable in black females, and increased in white males and females; these findings are worrying and warrant further monitoring. Furthermore, those with suppressed viral load had higher CD4 counts, highlighting the ongoing need for interventions to improve adherence during adolescence and transition. Age at transfer and having changed hospital at transfer had no effect on CD4 post-transition, but later calendar year of birth was associated with higher CD4. These findings lend support to improvements in transition planning in the UK in recent years, the individual approach to age at transition, and coordination of services across paediatric and adult care. However, the much higher proportion of young people with perinatally acquired HIV having a CD4 < 200 cells/mm3 in adult care, compared to other patients, is of great concern, and reasons for this require further investigation.
